# Concurrent diabetic ketoacidosis and pancreatitis in Paediatric acute lymphoblastic leukemia receiving L-asparaginase

**DOI:** 10.1186/s12887-020-02136-3

**Published:** 2020-05-18

**Authors:** Patel Zeeshan Jameel, Sham Lohiya, Amol Dongre, Sachin Damke, Bhavana B. Lakhkar

**Affiliations:** 1 Department of Paediatrics, Jawaharlal Nehru Medical College, Sawangi Meghe, Wardha, India; 2 Department of Oncology, Jawaharlal Nehru Medical College, Sawangi Meghe, Wardha, India

**Keywords:** Diabetic ketoacidosis, Pancreatitis, L-asparaginase

## Abstract

**Background:**

Although hyperglycemia and pancreatitis are known side effects of L-asparaginase, both contributing to the development of diabetic ketoacidosis (DKA) is unfamiliar in literature.

**Case presentation:**

We report a case of an adolescent girl, recently diagnosed with ALL, who presented with pain in abdomen and breathing difficulty following chemotherapy with L-asparaginase. On subsequent evaluation, she was found to have high anion gap metabolic acidosis, hyperglycemia and ketonuria. Ultrasonogram showed bulky pancreas. DKA was managed with fluid correction and insulin infusion. Pancreatitis was managed conservatively. She recovered completely with resolution of symptoms and without any major adverse events despite having such severe complications.

**Conclusion:**

We conclude that the combination of DKA and pancreatitis is a rare occurrence with significant morbidity and mortality. We recommend a close monitoring of blood glucose levels for hyperglycemia as well as a high index of clinical suspicion for pancreatitis in patients with ALL receiving L-asparaginase.

## Background

Acute lymphoblastic leukemia (ALL) accounts for approximately 77% cases of childhood leukemia [1]. In the year 2016, leukemia accounted for approximately 34,000 cases among all age groups in India [2]. The age-standardized incidence rate has declined sharply since 1990 by 16.1% but leukemia continues to be responsible for the highest proportion of the cancer DALYs (34.6%) in India among the age-group 0–14 years in the year 2016 [2]. The overall 5-year survival has increased to approximately 90% in the paediatric age group as a result of improvements in chemotherapy and risk stratification [1].

L-asparaginase has been a mainstay of pediatric chemotherapy protocols to treat patients with ALL since its discovery by Kidd in 1953 [3]. Standard chemotherapy regimens involved the use of vincristine and prednisolone to induce the first remission in patients with ALL until the discovery of L-asparaginase. The earliest literature describing the use of L-asparaginase in humans was reported in the mid-1960s by Dolowy et al. [4] and Hill et al. [5]. Treatment protocols with and without L-asparaginase have revealed that clinical outcomes were superior with those incorporating L-asparaginase [6]. Adverse effects of L-asparaginase include hypersensitivity reactions, including urticaria and anaphylaxis, thrombosis, hepatotoxicity, hypertriglyceridemia, hypoalbuminemia, intracranial hemorrhage, encephalopathy, myelosuppression, hyperglycemia, pancreatitis, and rarely diabetic ketoacidosis (DKA) [7–9].

Although, all of the above mentioned are known complications, DKA with pancreatitis in an adolescent receiving L-asparaginase presents a rare occurrence.

## Case presentation

A 14 year old adolescent female, belonging to central India, was diagnosed with B- cell ALL and started on induction phase of chemotherapy with Vincristine (1.5 mg/m^2^; once weekly), Prednisolone (60 mg/m^2^/day), Daunomycin (30 mg/m^2^; once weekly), intrathecal Methotrexate (12 mg; once weekly) and Non-pegylated L-asparaginase (10,000 IU/m^2^; on day 6,9,12,15,18 of induction phase). On the 21st day of the induction phase, patient complained of abdominal pain and breathlessness. Examination revealed tachycardia (150/min), tachypnea (40/min), a low volume, thready pulse and hypotension (BP-80/50 mmHg). She had cold extremities and signs of dehydration.

**Fig. 1:**
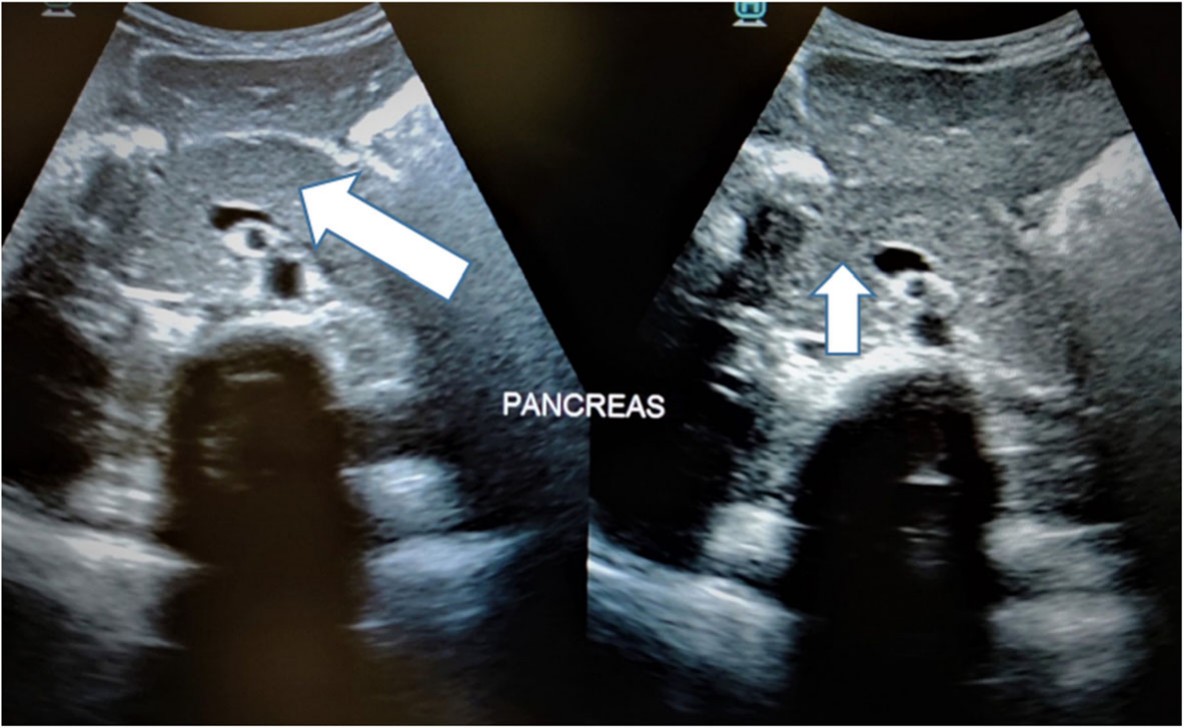
Grey scale ultrasound image showing features suggestive of acute pancreatitis: slightly bulky and enlarged visualized part of pancreas appearing uniformly hypoechoic. Main pancreatic duct appears normal. Minimal fat stranding was also noted

Investigations showed a blood sugar level of 501 mg/dl. Venous blood gas showed a pH of 7.22, pCO_2_ of 15.8 mmHg, HCO_3_^¯^ of 10.4 mmol/L, serum sodium of 138 mEq/L, serum potassium of 3.6 mEq/L, serum chloride of 110 mEq/L (Anion gap: 17.6). Urinary ketone bodies were positive (moderate size- 40 mg/dl). Further investigations showed, glycosylated HbA_1_C was 7.95%, serum amylase 479 IU/L and serum lipase 3340 IU/L. Liver enzymes and renal function tests were within normal limits. A sonogram of the abdomen showed bulky pancreas with normal echotexture (Fig. 1). To summarize, our patient had high anion gap metabolic acidosis, ketonuria and elevated levels of HbA_1_C, serum lipase and amylase with bulky pancreas on imaging. Based on above findings, a diagnosis of moderate DKA with acute pancreatitis was made.

She was managed with fluid resuscitation, insulin infusion in accordance with International Society for Pediatric and Adolescent Diabetes (ISPAD) 2018 regimen for DKA. Acute pancreatitis was managed conservatively. Metabolic acidosis resolved over the next 36 h following which insulin infusion was replaced by subcutaneous regular insulin, with the requirement of insulin being very low (0.15 IU/Kg/day). As this metabolic state was drug-induced and transient, she was continued on regular insulin till normalization of blood sugars. She was discharged with no further complications.

On follow-up, she is maintaining normal blood sugars without requiring insulin for the last few weeks. Further plan includes a bone marrow examination and continuation of chemotherapy excluding L-asparaginase.

## Discussion and conclusion

The present case constitutes a rare combination of DKA and pancreatitis, secondary to the use of L-asparaginase based chemotherapy during the induction phase.

The risk, among children receiving chemotherapy for ALL, of developing infections, hyperglycemia (attributable to the use of L-asparaginase and prednisolone), pancreatitis and the leukemic process itself is about 10–15% [10]. Plourde et al. [11] and Pui et al. [12] found the incidence of L-asparaginase use associated hyperglycemia to be 3.7 and 9.7% respectively. L-asparaginase causes hyperglycemia by depletion of asparagine resulting in a decline of insulin production. In addition, β cell dysfunction, caused due to pancreatitis, reduces insulin secretion from the beta cells of pancreas and hyperglucagonemia contribute to abnormal blood glucose levels [10, 13–15]. Risk factors for hyperglycemia during ALL therapy include age > 10 years, obesity, down’s syndrome, use of prednisolone and/or L-asparaginase [12]. Two of the above-mentioned risk factors were present in our case as well. The risk of hyperglycemia rises with the concomitant use of L-asparaginase and prednisolone; however, these episodes tend to be self-limiting and subside with no further complications [16, 17]. Additionally, pancreatitis itself may be responsible for metabolic abnormalities such as hyperglycemia and diabetes mellitus.

Despite hyperglycemia being commonly associated with L-asparaginase, DKA is a rare occurrence with a reported prevalence varying from 0.75–2.3% [10, 18, 19]. Cases in childhood have been reported of DKA in ALL associated with L-asparagianase [18–22]. There is no conclusive evidence on the time taken for diabetogenic effects of L-asparaginase [10]. In our case, the patient developed DKA on 16th day following the administration of first dose of the L-asparaginase in the induction phase. DKA is generally mild with short term morbidity but may lead to permanent sequelae. Wolthers et al. [23] described in their population-based Nordic Society of Paediatric Hematology and Oncology (NOPHO) 2008 ALL pancreatitis study that 8% (7/86) of children, who had developed pancreatitis during the course of treatment for ALL, required insulin therapy on follow-up.

The second complication encountered in our patient was acute pancreatitis. In a recent study evaluating children with ALL, the incidence of L-asparaginase induced pancreatitis was 6.8% (86/1285) [24] while other studies have reported from 2 to 18% [7, 25–27]. In spite of pancreatitis being a known complication of L-asparaginase, the exact pathogenesis is yet to be discovered. A possible mechanism could be that it decreases the protein synthesis due to systemic depletion of asparagine [28]. Multiple studies have shown the possible role of genes such as *ULK2* and trypsin encoding *PRSS1-PRSS2* [29, 30]. Although DKA causing elevation of pancreatic enzymes is a known entity, clinical pancreatitis is rare. Incidence of acute pancreatitis in children with DKA was reported to be 2% [31]. The mechanism by which DKA causes pancreatitis still remains elusive. It has been postulated that minor injury or inflammation in pancreas leads to leakage of pancreatic enzymes [32]. In addition, hyperlipidemia contributes to acute pancreatitis by build-up of degradation products from triglycerides due to the action of pancreatic lipases, which in turn are directly toxic to the acinar cells [32, 33]. In a systematic review, it was concluded that older age, formulation of L-asparaginase, higher ALL risk stratification and a higher dosing of L-asparagine appear to raise the risk of pancreatitis in some studies [34]. In a retrospective cohort study by Treepongkaruna et al. [27], it was reported that pancreatitis developed after a median of 5.5 doses with a median interval of 4 days from the last dose of L-asparaginase therapy. Our patient showed signs of pancreatitis 2 days after receiving 5th dose. The clinical course of both complications can range from mild to severe disease. The mortality is significantly higher in ALL patients with acute pancreatitis (43.8% Vs 19.3%) [27].

There is a possibility that combined use of L-asparaginase and prednisolone along with L-asparaginase induced pancreatitis might have precipitated ketoacidosis in our patient. However, it is difficult to ascertain if the primary inciting event was pancreatitis or diabetic ketoacidosis. Prompt diagnosis and appropriate management of both DKA and pancreatitis were extremely crucial for the survival of our patient.

From this case we infer that DKA and pancreatitis occurring together in a patient with ALL on chemotherapy is rare but when it does occur, is associated with significant morbidity and mortality. In the absence of a consensus on the frequency of blood glucose monitoring, we recommend for a close follow-up of blood glucose levels for hyperglycemia as well as a high index of clinical suspicion for pancreatitis is essential in patients with ALL receiving L-asparaginase.

### Learning points

- Simple, minimally invasive blood tests such as blood glucose level can detect impaired glucose metabolism at an earlier stage, thus, preventing progression to complications such as DKA.

## Data Availability

The datasets used and/or analysed during the current study are available from the corresponding author on reasonable request.

## Abbreviations

ALL: Acute lymphoblastic leukemia
DKA: Diabetic ketoacidosis
ISPAD: International Society for Pediatric and Adolescent Diabetes
NOPHO: Nordic Society of Paediatric Hematology and Oncology
DALYs: Disability adjusted life year
